# Incidence of contrast-associated acute kidney injury differs by procedure type in neuroendovascular surgery

**DOI:** 10.1177/19714009261473175

**Published:** 2026-07-31

**Authors:** Yaxiaerjiang Yalikun, Masakazu Hayashida, Masataka Fukuda, Atsuko Hara, Chieko Mitaka, Izumi Kawagoe

**Affiliations:** 1 Department of Anesthesiology and Pain Medicine, Juntendo University Graduate School of Medicine, Tokyo, Japan

**Keywords:** aneurysmal subarachnoid hemorrhage (aSAH), carotid artery stenosis (CAS), contrast-associated acute kidney injury (CA-AKI), creatinine, neuroendovascular surgery (NES)

## Abstract

**Background:**

Evidence regarding the incidence and risk factors of contrast-associated acute kidney injury (CA-AKI) after neuroendovascular surgery (NES) remains limited. This study aimed to identify risk factors for CA-AKI after NES.

**Methods:**

We retrospectively analyzed adolescent and adult patients who underwent NES under general anesthesia at our institution between 2014 and 2024. CA-AKI was defined as a serum creatinine increase ≥0.3 mg/dL within 48 h, or ≥1.5 times baseline within 7 days. Risk factors were assessed using logistic regression.

**Results:**

Among 2412 patients (840 males) aged 10–91 years, NES indications included unruptured cerebral aneurysm (UCA, *n* = 1808), carotid artery stenosis (CAS, *n* = 212), dural arteriovenous fistula (DAVF, *n* = 183), aneurysmal subarachnoid hemorrhage (aSAH, *n* = 107), and arteriovenous malformation (AVM, *n* = 102). The overall incidence of CA-AKI was 1.91%. By lesion type, incidences were 9.35% (aSAH), 9.91% (CAS), 2.94% (AVM), 0.55% (DAVF), and 0.61% (UCA). Multivariate analysis identified diabetes mellitus (odds ratio [OR], 2.414; 95% confidence interval [CI], 1.099–5.306; *p* = .028), lower preoperative total protein (OR, 0.447; 95% CI, 0.265–0.754; *p* = .003), higher preoperative creatinine (OR, 5.692; 95% CI, 2.962–10.938; *p* = <0.001), and lesion type (aSAH vs. UCA: OR, 17.672; CAS vs. UCA: OR, 9.551; both *p* < .001) as significant predictors.

**Conclusion:**

Patients undergoing NES for aSAH or CAS were associated with an increased risk of CA-AKI, independent of known risk factors. To our knowledge, this is the first study to demonstrate lesion-specific differences in CA-AKI risk after NES.

## Introduction

Neuroendovascular surgery (NES) is less invasive than open craniotomy, and its use has increased over time while open procedures have declined.^1^ However, NES requires contrast agents, which carry a risk of contrast-associated acute kidney injury (CA-AKI).^2–4^ Contrast agents exert direct tubular toxicity and can induce renal ischemia via vasoactive mechanisms.^4^

Extensive literature exists on CA-AKI after percutaneous coronary interventions (PCIs), with reported risk factors including hemodynamic instability, advanced age, anemia, chronic kidney disease, diabetes mellitus, hypertension, and high contrast dose.^2,3^ For NES, most available data concern acute ischemic stroke: a meta-analysis reported a 5.0% incidence of CA-AKI (95% confidence interval [CI], 2.1%–8.9%) across 15 studies involving 27246 patients.^5^ In contrast, studies on CA-AKI after NES for other cerebrovascular lesions, such as cerebral aneurysms (CA), remain scarce, and risk factors are not well defined.^6,7^

At our institution, NES for lesions other than ischemic stroke is performed by neurosurgeons under general anesthesia in the operating room, mainly for treatment of unruptured CA (UCA), arteriovenous malformation (AVM), dural arteriovenous fistula (DAVF), carotid artery stenosis (CAS), and aneurysmal subarachnoid hemorrhage (aSAH). By contrast, endovascular stroke procedures are performed by neurologists under local anesthesia in the radiology department. To date, only one study each has reported CA-AKI incidence after NES for UCA^8^ and aSAH,^9^ and five studies have addressed CAS.^10–14^ No studies have investigated CA-AKI after NES for AVM or DAVF. A prior study suggested—but did not confirm—that NES for CAS may carry a higher risk than other NES procedure types.^7^ However, no study has directly compared CA-AKI incidence across different NES procedure types.

Accordingly, the present study was designed to compare the incidence of CA-AKI across different NES procedure types and to identify risk factors associated with its occurrence.

## Methods

### Study design and patients

We retrospectively analyzed adolescent and adult patients aged ≥10 years (per World Health Organization criteria) who underwent elective or emergency NES (coiling and/or stenting) that were performed by neurosurgeons under general anesthesia in the operation room at Juntendo University Hospital between March 2014 and February 2024. The study was conducted in accordance with the Declaration of Helsinki. The study protocol was reviewed and approved by the Ethics Committee of Juntendo University Hospital (protocol code: E24-0322; approval date: 29 October 2024). Informed consent was waived due to the retrospective design of the study.

Eligible patients were identified using the ORSYS^®^ electronic anesthesia record system (Philips Japan, Tokyo, Japan), which contains records from procedures performed in the operating room. Consequently, patients undergoing thrombectomy for acute ischemic stroke were not included because these procedures were performed by neurologists under local anesthesia in the radiology department outside the operating room.

The following patients were excluded: patients under 10 years old; patients with end-stage chronic kidney disease who were receiving dialysis; patients who only had diagnostic angiography; patients with missing perioperative creatinine data; and patients who underwent rarely performed NES procedures.

All elective patients received intravenous saline hydration at a rate of 62.5 mL/h (1500 mL/day) from 16:00 on the day before surgery until anesthesia. Patients were not premedicated before anesthesia. NES procedures were performed under sevoflurane-based general anesthesia, using a nonionic low-osmolar contrast medium (Iomeprol®, Bracco-Eizai, Tokyo, Japan).

### Data collection

Collected data included demographics (age, sex, and body size), comorbidities (hypertension, diabetes mellitus, and cardiac disease), laboratory variables (hemoglobin, total protein, creatinine, and estimated glomerular filtration rate [eGFR]), and operative variables (surgical indication, elective/emergency status, surgical and anesthesia durations, and contrast volume). Laboratory data were obtained preoperatively and on postoperative days 1 through 3.

### Definitions

CA-AKI was defined according to the Kidney Disease: Improving Global Outcomes (KDIGO) criteria as an increase in serum creatinine of ≥0.3 mg/dL within 48 h, or ≥1.5 times the baseline value within 7 days.^15^ The urine output criterion was not applied because data were insufficient in a part of patients.

### Statistical analysis

Continuous variables are presented as medians with interquartile ranges (IQRs) due to non-normal distributions, and categorical variables are presented as numbers and percentages. Comparisons between two groups were conducted using the Mann–Whitney *U* test or the chi-square test. Comparisons among multiple procedure types were performed using the Kruskal–Wallis test or the chi-square test, followed by pairwise comparisons with the Mann–Whitney *U* test or the chi-square test applying Bonferroni correction. Correlation between two variables was examined with Spearman’s correlation analysis. Logistic regression analysis was used to identify predictors of CA-AKI. All statistical analyses were performed using SPSS version 29 (IBM, Armonk, NY, USA), and a *p* value <.05 was considered statistically significant unless otherwise specified.

## Results

**Figure 1.**
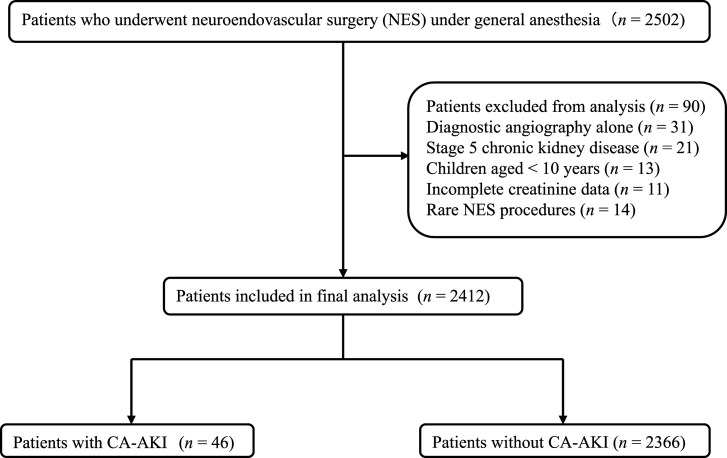
Flow chart of patient selection and classification by contrast-associated acute kidney injury (CA-AKI) status.

We reviewed records of 2502 patients. Initially, 76 patients were excluded: 31 who underwent diagnostic angiography alone, 21 with end-stage chronic kidney disease on dialysis, 13 aged <10 years, and 11 with incomplete creatinine data (Figure 1). Among the remaining 2426 patients, vascular lesions treated with NES included UCA (*n* = 1808, including 10 unruptured dissecting aneurysms [DA]), CAS (*n* = 212, including three subclavian artery stenoses and one vertebral artery stenosis), DAVF (*n* = 183), aSAH (*n* = 107; 106 due to ruptured CA and one due to ruptured DA), AVM (*n* = 102), tumors (*n* = 10), and cerebral thromboembolism (*n* = 4). Because no CA-AKI occurred in the rare tumor or thromboembolism cases, these 14 patients were subsequently excluded. Finally, data from 2412 patients (840 males, aged 10–91 years; including 13 adolescents aged 10–19 years) were analyzed (Figure 1).

Clinical characteristics of the 2412 patients are summarized in Table 1. Based on baseline eGFR, 18 (0.75%), 72 (2.99%), 234 (9.70%), and 2088 (86.57%) patients had severe, moderate, mild, and no chronic kidney disease, defined as eGFR <30, 30–45, 45–60, and ≥60 mL/min/1.73 m^2^, respectively. The contrast medium volume was 180 (150–220) mL (median [IQR]) or 192.8 ± 72.1 mL (mean ± standard deviation).

**Table 1.** Baseline clinical characteristics of the total cohort and of patients with and without contrast-associated acute kidney injury (CA-AKI).

Group	Total cohort	CA-AKI	No CA-AKI	*p* value
Number (%) of patients	2412 (100%)	46 (1.91%)	2366 (98.09%)	
Demographics				
Age (years)	63 (53–73)	73 (59–78)	63 (53–73)	<0.001
Gender, male (*n* (%))	840 (34.8%)	27 (58.7%)	813 (34.4%)	<0.001
Body height (cm)	158.5 (153–165.2)	162.8 (158.2–167)	158.3 (153–165.1)	0.036
Body weight (kg)	57.4 (50.9–66.8)	65.0 (55.3–69.5)	57.3 (50.85–66.5)	0.015
Body mass index (kg/m^2^)	23.0 (20.7–25.4)	24.0 (22.3–25.9)	23.0 (20.7–25.3)	0.027
Comorbidities				
Diabetes mellitus (*n* (%))	250 (10.4%)	18 (39.1%)	232 (9.8%)	<0.001
Hypertension (*n* (%))	1096 (45.4%)	29 (63.0%)	1067 (45.1%)	0.015
Cardiac disease (*n* (%))	207 (8.6%)	10 (21.7%)	197 (8.3%)	0.005
Preoperative laboratory test data				
Hemoglobin (g/dL)	13.2 (12.4–14.2)	13.45 (11.7–14.5)	13.2 (12.4–14.2)	0.943
Total protein (g/dL)	7.0 (6.7–7.3)	6.8 (6.5–7.1)	7.0 (6.7–7.3)	0.005
Serum creatinine (mg/dL)	0.63 (0.53–0.76)	0.875 (0.68–1.34)	0.63 (0.53–0.76)	<0.001
eGFR (mL/min/1.73 m^2^)	80.65 (67.9–94.1)	59.25 (41.0–84.7)	80.8 (68.3–94.2)	<0.001
Surgical and anesthetic factors				
Emergency surgery (*n* (%))	138 (5.7%)	10 (21.7%)	128 (5.4%)	<0.001
Duration of surgery (min)	81 (58–115)	81.5 (53–130)	81 (58–115)	0.973
Duration of anesthesia (min)	113 (88–150)	110.5 (82–172)	113 (88–150)	0.774
Contrast volume (mL/kg)	3.15 (2.41–4.00)	2.92 (2.23–3.88)	3.15 (2.41–4.00)	0.161
Lesions treated with neuroendovasular surgery (NES)				
aSAH (*n* (%))	107 (4.4%)	10 (21.7%)	97 (4.1%)	<0.001
CAS (*n* (%))	212 (8.8%)	21 (45.7%)	191 (8.1%)	​
AVM (*n* (%))	102 (4.2%)	3 (6.5%)	99 (4.2%)	​
DAVF (*n* (%))	183 (7.6%)	1 (2.2%)	182 (7.7%)	​
UCA (*n* (%))	1808 (75.0%)	11 (23.9%)	1797 (76.0%)	​

Data are presented as medians (interquartile ranges) or numbers (percentages). Comparisons between patients with and without CA-AKI were made using the Mann–Whitney *U* test or the chi-square test.

aSAH: aneurysmal subarachnoid hemorrhage; AVM: arteriovenous malformation; CAS: carotid artery stenosis; DAVF: dural arteriovenous fistula; eGFR: estimated glomerular filtration rate; UCA: unruptured cerebral aneurysm.

### Clinical background data in the five NES groups

All baseline variables differed significantly among the groups (Table 2). Briefly, CAS patients were the oldest, had the highest proportion of males, showed the greatest prevalences of hypertension, diabetes mellitus, and cardiac disease (mainly coronary artery disease), exhibited the highest preoperative creatinine levels and the lowest preoperative eGFR levels, and received the smallest contrast volume. In contrast, AVM patients were the youngest, showed the smallest comorbidity prevalences, exhibited the lowest creatinine levels and the highest eGFR levels, and received the largest contrast volume. Surgery and anesthesia durations were shortest in CAS patients and longest in DAVF patients. UCA patients had the highest proportion of females and the highest preoperative total protein levels.

**Table 2.** Clinical characteristics and incidences of contrast-associated acute kidney injury (CA-AKI) in the five neuroendovascular surgery (NES) procedure groups.

NES procedure group	aSAH (*n* = 107)	CAS (*n* = 212)	AVM (*n* = 102)	DAVF (*n* = 183)	UCA (*n* = 1808)	*p* value
Outcome						
CA-AKI (*n* (%))	10 (9.35%)*†	21 (9.91%)*†	3 (2.94%)	1 (0.55%)§||	11 (0.61%)§||	<0.001
Demographics						
Age (years)	65 (51–74.5)†‡§	72.5 (67–77)*‡||	49.5 (32–61)*†§||	70 (60.5–76)*‡||	61 (52–71)†‡§	<0.001
Gender, male (*n* (%))	34 (31.8%)§	165 (77.8%)*†‡||	49 (48.0%)*§	86 (47.0%)*§	506 (28.0%)†‡§	<0.001
Body height (cm)	157.3 (151–166)§	164.55 (159.75–170)*†‡||	160.8 (156–168)*§	159.5 (152–169)§	157.45 (152.5–163.95)‡§	<0.001
Body weight (kg)	55 (50–65.2)†§	64.3 (56.85–71.15)*‡||	55.1 (52–65.5)§	60.5 (53.2–69.5)*||	56.5 (50.1–65.7)†§	<0.001
Body mass index (kg/m^2^)	22.6 (19.85–24.9)†	23.5 (21.85–25.35)‡	21.75 (20.2–25.0)†§	23.8 (21.6–26.1)*‡||	22.9 (20.6–25.3)†	<0.001
Comorbidities						
Diabetes mellitus (*n* (%))	11 (10.3%)§	92 (43.4%)*†‡||	7 (6.9%)§	28 (15.3%)*§	112 (6.2%)†§	<0.001
Hypertension (*n* (%))	23 (21.5%)*†§	135 (63.7%)*†‡||	22 (21.6%)*†§	72 (39.3%)‡§||	844 (46.7%)‡§||	<0.001
Cardiac disease (*n* (%))	4 (3.7%)§	77 (36.3%)*†‡||	2 (2.0%)§	15 (8.2%)§	109 (6.0%)§	<0.001
Preoperative laboratory test data						
Hemoglobin (g/dL)	13.3 (12.1–14.3)	13.2 (12.2–14.3)	13.3 (12.1–14.3)	13.6 (12.6–14.7)*	13.2 (12.4–14.1)†	0.028
Total protein (g/dL)	6.9 (6.55–7.3)	6.9 (6.6–7.2)*	6.7 (6.4–7.1)*	6.9 (6.6–7.2)*	7.0 (6.8–7.3)†‡§	<0.001
Serum creatinine (mg/dL)	0.62 (0.505–0.76)§	0.79 (0.685–0.965)*†‡||	0.60 (0.49–0.77)†§	0.67 (0.57–0.81)*‡§	0.61 (0.53–0.73)†§	<0.001
eGFR (mL/min/1.73 m^2^)	83.7 (65.25–100.9)‡§	70.3 (58.55–84.0)*†‡||	95.0 (82.9–110.5)†§||	77.0 (66.75–91.65)*‡§	81.1 (69.2–93.8)†§	<0.001
Surgical and anesthetic data						
Emergency surgery (*n* (%))	107 (100%)*†‡§	4 (1.9%) ||	7 (6.9%)*||	8 (4.4%)*||	12 (0.7%)†‡||	<0.001
Duration of surgery (min)	86 (67.5–121.5)†‡§	60 (48–78)*†‡||	124 (91–177)*§||	152 (106–207)*§||	79 (58–107)†‡§	<0.001
Duration of anesthesia (min)	134 (107.5–171)*†‡§	85 (70.5–104.5)*†‡||	156.5 (121–207)*§||	186 (141–238)*§||	109 (88–141)†‡§||	<0.001
Contrast volume (mL)	190 (170–210)§	180 (140–200)†‡||	220 (150–260)*§	210 (150–260)*§	180 (150–220)†‡	<0.001

Data are presented as medians (interquartile ranges) or numbers (percentages). Comparisons among the five NES procedure groups were conducted using the Kruskal–Wallis test or the chi-square test, with *p* values reported in the table. When significant differences were found, pairwise comparisons were performed using the Mann–Whitney *U* test or the chi-square test with Bonferroni correction.

*p < .005 versus UCA; †*p* < .005 versus DAVF; ‡*p* < .005 versus AVM; §*p* < .005 versus CAS; ||*p* < .005 versus aSAH (Bonferroni correction).

aSAH: aneurysmal subarachnoid hemorrhage; AVM: arteriovenous malformation; CAS: carotid artery stenosis; DAVF: dural arteriovenous fistula; eGFR: estimated glomerular filtration rate; UCA: unruptured cerebral aneurysm.

Emergency NES was performed in 138 patients (5.7% overall), including 100% of aSAH, 6.9% of AVM, 4.4% of DAVF, 1.9% of CAS, and 0.7% of UCA patients (Table 2).

### Incidence of CA-AKI

Twenty-seven patients met only the KDIGO absolute criterion (an increase in serum creatinine of ≥0.3 mg/dL within 48 h), five met only the ratio criterion (an increase to ≥1.5 times the baseline value within 7 days), and fourteen met both criteria. All these serum creatinine increases that met the criteria occurred by the morning of Postoperative Day 3. Overall, CA-AKI occurred in 46 of 2412 patients (1.91%) (Table 1).

By lesion type, incidence rates were 9.35% for aSAH, 9.91% for CAS, 2.94% for AVM, 0.55% for DAVF, and 0.61% for UCA (Table 2). Incidences were significantly higher in aSAH and CAS than in DAVF and UCA (Bonferroni-adjusted *p* < .005) (Table 2).

Notably, all CA-AKI events after emergency NES (*n* = 10) occurred exclusively in aSAH patients. The overall incidence was higher after emergency than elective NES (7.25% [10/138] vs. 1.58% [36/2274], *p* < .001).

Among 107 patients with aSAH, neither initial Glasgow Coma Scale (GCS) score nor Fisher grade differed between patients with and without CA-AKI (*p* = .689 and *p* = .241, respectively; chi-square test) (Table 3). Consistently, neither severe neurological impairment (initial GCS score ≤8; *n* = 11 [10.3%]) nor Fisher grade 4 (*n* = 14 [13.1%]) was associated with CA-AKI development (*p* = .976 and *p* = .762, respectively; logistic regression analysis). Glasgow Outcome Scale (GOS) scores also did not differ between patients with and without CA-AKI (*p* = .168; chi-square test) (Table 3). However, in-hospital mortality (GOS 1; *n* = 6) was significantly associated with CA-AKI (30.0% [3/10] vs. 3.1% [3/97], *p* < .001 by chi-square test and *p* = .004 by logistic regression analysis).

**Table 3.** Glasgow Coma Scale (GCS) score, Fisher grade, and Glasgow Outcome Scale (GOS) in the overall aneurysmal subarachnoid hemorrhage (aSAH) cohort and according to contrast-associated acute kidney injury (CA-AKI) status.

​	Overall aSAH cohort (*n* = 107)	CA-AKI (*n* = 10)	No CA-AKI (*n* = 97)	*p* value
Glasgow Coma Scale (GCS) score				
3	2 (1.9%)	0 (0.0%)	2 (2.1%)	0.689
4	1 (0.9%)	0 (0.0%)	1 (1.0%)	
6	3 (2.8%)	0 (0.0%)	3 (3.1%)	
7	3 (2.8%)	1 (10.0%)	2 (2.1%)	
8	2 (1.9%)	0 (0.0%)	2 (2.1%)	
9	4 (3.7%)	0 (0.0%)	4 (4.1%)	
10	1 (0.9%)	0 (0.0%)	1 (1.0%)	
11	3 (2.8%)	2 (20.0%)	1 (1.0%)	
12	1 (0.9%)	0 (0.0%)	1 (1.0%)	
13	14 (13.1%)	0 (0.0%)	14 (14.4%)	
14	21 (19.6%)	3 (30.0%)	18 (18.6%)	
15	52 (48.6%)	4 (40.0%)	48 (49.5%)	
Fisher grade				
1	23 (21.5%)	0 (0.0)	23 (23.7)	0.241
2	19 (17.8%)	2 (20.0)	17 (17.5)	
3	51 (47.7%)	7 (70.0)	44 (45.4)	
4	14 (13.1%)	1 (10.0)	13 (13.4)	
Glasgow Outcome Scale (GOS)				
1	6 (5.6%)	3 (30.0%)	3 (3.1%)	0.168
2	4 (3.7%)	0 (0.0%)	4 (4.1%)	
3	6 (5.6%)	0 (0.0%)	6 (6.2%)	
4	22 (20.6%)	2 (20.0%)	20 (20.6%)	
5	69 (64.5%)	5 (50.0%)	64 (66.0%)	

Data are presented as numbers (percentages). Data were compared between aSAH patients with and without CA-AKI using the chi-square test.

In contrast, among 36 patients who developed CA-AKI after NES for lesions other than aSAH, serum creatinine elevations resolved within several days, and no in-hospital deaths occurred.

### Comparison of patients with and without CA-AKI

When stratified by CA-AKI status, patients who developed CA-AKI were older, more frequently male, and had larger body size. They also had lower preoperative total protein levels, higher preoperative creatinine levels, and lower preoperative eGFR levels, and greater prevalences of hypertension, diabetes mellitus, and cardiac disease (mainly coronary artery disease) (all *p* < .05) (Table 1). In addition, emergency surgery and lesion type differed significantly between the groups (both *p* < .001) (Table 1).

### Associations with CA-AKI

In univariate logistic regression, older age, male sex, larger body weight, hypertension, diabetes mellitus, cardiac disease, lower total protein, higher creatinine, lower eGFR, emergency surgery, and lesion type (aSAH, CAS, and AVM vs. UCA) were significantly associated with CA-AKI (all *p* < .05; Table 4).

Because of the limited number of CA-AKI events (*n* = 46), only variables significant in univariate analysis (*p* < .05) were included in multivariate analysis. However, emergency surgery was excluded from multivariate analysis due to quasi-complete separation with aSAH, since all 10 CA-AKI events following emergency surgery occurred exclusively in patients with aSAH, while none occurred among the 31 emergency surgery patients with other vascular lesions. Quasi-complete separation occurs when at least one cell of a 2 × 2 table between a binary predictor and a binary outcome contains zero observations, thereby preventing the stable solution of logistic regression analysis.^16^ In addition, eGFR was excluded because it was strongly correlated with serum creatinine (ρ = −0.809, *p* < .001), and univariable logistic regression demonstrated inferior model performance for eGFR compared with serum creatinine, as indicated by a lower Nagelkerke’s R^2^ (0.070 vs. 0.117) and a lower area under the receiver operating characteristic curve (AUC; 0.701 vs. 0.738).

**Table 4.** Univariate and multivariate associations between clinical variables and the development of contrast-associated acute kidney injury (CA-AKI).

Clinical variables	Univariate logistic regression		Multivariate logistic regression	
	OR (95% CI)	*p* value	OR (95% CI)	*p* value
Demographics				
Age (years)	1.043 (1.017–1.070)	0.001	1.005 (0.975–1.036)	0.761
Males (*n* (%))	2.715 (1.500–4.912)	<0.001	0.770 (0.347–1.707)	0.519
Body height (cm)	1.024 (0.993–1.056)	0.137	N/A*	​
Body weight (kg)	1.022 (1.001–1.045)	0.044	1.007 (0.977–1.037)	0.668
Body mass index (kg/m^2^)	1.056 (0.983–1.135)	0.134	N/A*	​
Comorbidities				
Hypertension (*n* (%))	2.077 (1.135–3.800)	0.018	1.749 (0.807–3.791)	0.156
Diabetes mellitus (*n* (%))	5.913 (3.221–10.855)	<0.001	2.414 (1.099–5.306)	0.028
Cardiac disease (*n* (%))	3.058 (1.495–6.256)	0.002	0.627 (0.253–1.554)	0.313
Preoperative blood test data				
Hemoglobin (g/dL)	0.967 (0.794–1.178)	0.739	N/A*	​
Total protein (g/dL)	0.392 (0.251–0.612)	<0.001	0.447 (0.265–0.754)	0.003
Serum creatinine (mg/dL)	7.932 (4.183–15.042)	<0.001	5.692 (2.962–10.938)	<0.001
eGFR (mL/min/1.73 m^2^)	0.962 (0.948–0.976)	<0.001	N/A**	​
Surgical and anesthetic data				
Emergency surgery (*n* (%))	4.857 (2.357–10.007)	<0.001	N/A***	​
Duration of surgery (min)	1.001 (0.995–1.006)	0.831	N/A*	​
Duration of anesthesia (min)	1.001 (0.997–1.006)	0.578	N/A*	​
Contrast volume (mL/kg)	0.839 (0.661–1.065)	0.149	N/A*	​
Lesions treated with neuroendovascular surgery (NES)				
aSAH (*n* (%))	16.842 (6.983–40.620)	<0.001	17.672 (6.691–46.673)	<0.001
CAS (*n* (%))	17.961 (8.530–37.819)	<0.001	9.551 (3.668–24.870)	<0.001
AVM (*n* (%))	4.950 (1.359–18.029)	0.015	3.171 (0.620–16.225)	0.166
DAVF (*n* (%))	0.918 (0.115–6.992)	0.918	0.394 (0.039–4.020)	0.432
UCA (*n* (%))	1 (reference)	​	1 (reference)	​

Data are presented as medians (interquartile ranges) or numbers (percentages). Results of univariate and multivariate logistic regression analyses are shown as odds ratios (ORs) with 95% confidence intervals (95% CIs).

*Excluded from a multivariate model because of a *p* value >.05 in univariate analysis, ** excluded from the model because of a strong correlation with serum creatinine (ρ = −0.809, *p* < .001), and *** excluded from the model because of quasi-complete separation with aSAH.

aSAH: aneurysmal subarachnoid hemorrhage; AVM: arteriovenous malformation; CAS: carotid artery stenosis; DAVF: dural arteriovenous fistula; eGFR: estimated glomerular filtration rate; UCA: unruptured cerebral aneurysm.

Consequently, multivariate logistic regression analysis was performed using age, sex, body weight, hypertension, diabetes mellitus, cardiac disease, total protein, creatinine, and vascular lesion type as covariates. The analysis revealed that diabetes mellitus (*p* = .026), lower total protein (*p* = .003), higher creatinine (*p* < .001), and lesion type (aSAH vs. UCA, *p* < .001; CAS vs. UCA, *p* < .001) were independent predictors of CA-AKI after NES (Table 4).

## Discussion

In this study, diabetes mellitus, lower total protein, higher creatinine, and NES procedures for aSAH and CAS were independently associated with an increased risk of CA-AKI.

To our knowledge, this is the first study to directly compare CA-AKI incidence across multiple NES procedure types. Previous reports have described overall CA-AKI rates after NES^6,7^ or after specific procedures, including acute ischemic stroke,^5^ UCA,^8^ aSAH,^9^ and CAS,^10–14^ whereas incidence following NES for AVM or DAVF has not been reported. Our findings demonstrate that CA-AKI risk varies significantly by lesion type, although patients undergoing NES for acute ischemic stroke were not included in our cohort.

As suggested by a previous study,^7^ the incidence of CA-AKI after NES for CAS was relatively high at 9.91%. Prior reports have described rates ranging from 1.7% to 34.0%,^10–14^ with a pooled incidence of 18.5% (216/1167). Patients in our CAS group were characterized by the oldest age, the highest baseline creatinine, the lowest baseline eGFR, and the highest prevalences of hypertension, diabetes mellitus, and cardiac disease—all recognized CA-AKI risk factors in PCI patients.^2,3^ Several pathophysiological mechanisms may further explain this elevated risk. Carotid atherosclerosis is associated with reduced renal function even in adults without known kidney disease,^17^ and severe CAS is often accompanied by renal artery stenosis and impaired renal function.^18^ Conversely, carotid atherosclerosis is common in patients with chronic kidney disease,^19^ and its severity correlates with diabetic microangiopathy (retinopathy and nephropathy) in diabetic populations.^20^ In addition, pre-existing systemic inflammation may contribute to CA-AKI development in CAS patients.^13^ Furthermore, hypotension and bradycardia induced by carotid sinus reflex during NES for CAS may exacerbate renal vulnerability.^10–12^ Notably, even brief hypotension for 2.5 min may be associated with increased CA-AKI risk in CAS patients with chronic kidney disease.^11^ Taken together, comorbidities and procedure-related hemodynamic instability likely contribute to the particularly high risk of CA-AKI after NES for CAS.

In this study, a high CA-AKI incidence was also observed in aSAH patients (9.35%). Only one prior study of NES for aSAH has reported a rate of 7.3%.^9^ Outside the NES setting, AKI rates in SAH patients are even higher, ranging from 12% to 38% in neurocritical care cohorts.^21^ Similarly, four studies of aSAH patients treated with surgical clipping reported AKI incidences of 6.2–34.0%,^22–25^ with a pooled rate of 12.8% (118/922). The elevated risk in aSAH may partly reflect limited preoperative hydration in emergencies, although in our cohort, CA-AKI did not occur after emergency NES for other lesions. Additional contributors may include repeated contrast exposure, perioperative fluid imbalance (both hypovolemia and hypervolemia), hemodynamic instability including neurogenic shock,^26,27^ sympathetic overactivation (catecholamine surge) leading to renal vasoconstriction,^28^ and severe systemic inflammation triggered by aSAH.^29^ Our findings extend previous evidence by demonstrating that aSAH carries a high CA-AKI risk even in the NES setting.

A previous study reported a 7.3% incidence of CA-AKI after NES for aSAH and identified a severe initial GCS score (≤8) as a significant risk factor.^9^ In contrast, the initial GCS score was not associated with CA-AKI in the present study. This discrepancy may reflect differences in aSAH severity, as patients with an initial GCS score ≤8 (10.3% vs. 21.9%) or Fisher Scale Grade 4 (13.1% vs. 45.8%) were less frequent in the present study, compared with the previous study.^9^ Nevertheless, the incidence of CA-AKI after NES for aSAH remained relatively high (9.35%), suggesting that NES for aSAH, or aSAH per se, may increase the risk of AKI regardless of clinical severity. Importantly, CA-AKI was associated with increased mortality in patients with aSAH, consistent with previous findings,^9^ whereas no death occurred among patients who developed CA-AKI after NES for non-aSAH lesions.

CA-AKI rates after NES for AVM (2.94%), DAVF (0.55%), and UCA (0.61%) were relatively low, with no significant differences after adjustment. Prior work has shown that with adequate hydration and limited contrast use, the risk of CA-AKI after NES or cerebral angiography can be minimized (0.54%) even in patients with chronic kidney disease.^6^ Although a single prior study investigating CA-AKI after NES for UCA reported a relatively higher incidence (5.6%),^8^ variability across studies likely reflects differences in diagnostic criteria, study design, patient comorbidities, and perioperative management—similar to the wide ranges observed in CAS and aSAH cohorts.^10–14,21–25^ Further studies are warranted to better define AKI risk by NES procedure type.

The overall incidence of CA-AKI after NES in the present study (1.9%) was substantially lower than that reported after PCI (6.4–7.3%), despite a similar mean contrast volume (192.8 mL in our cohort vs. approximately 190–200 mL in large PCI studies).^3,30,31^ Consistent with previous studies of NES,^5–13^ contrast volume was not significantly associated with CA-AKI in our cohort, in contrast to PCI cohorts showing the significant association.^2,3,30,31^ One possible explanation for these differences is the routine use of preprocedural intravenous hydration in all elective NES patients. Historically, routine hydration was commonly performed in patients undergoing PCI^2^; however, subsequent guidelines and reviews have restricted prophylactic hydration to patients with impaired renal function or other grave risk factors.^4^ Because our study was retrospective and lacked a control group managed according to contemporary guideline-directed hydration strategies, the independent effect of routine prehydration cannot be determined. Nevertheless, previous studies in elective PCI have reported no reduction in the CA-AKI incidence with routine intravenous prehydration in unselected patients,^32,33^ suggesting that our hydration protocol alone is unlikely to fully explain the low incidence of CA-AKI and no effect of contrast volume on CA-AKI in our NES cohort.

Another explanation is that contrast volume may contribute less to CA-AKI risk than underlying patient characteristics. In the original Mehran risk model, multiple clinical variables, including hemodynamic instability, chronic kidney disease, and diabetes mellitus, showed much stronger associations with CA-AKI than contrast volume.^2^ More recent analyses have similarly demonstrated that baseline patient risk exerts a substantially greater influence on CA-AKI development than variations in contrast volume.^30,31^ While presentation of an ST-segment elevation myocardial infarction, cardiogenic shock, congestive heart failure, and severe chronic kidney disease (eGFR <30 mL/min/1.73 m^2^) are the strongest predictors for CA-AKI in PCI patients,^2,3^ our NES cohort contained fewer patients with severe chronic kidney disease compared with PCI populations^3,30,31^ and lacked major hemodynamic risk factors associated with myocardial ischemia. Consequently, NES patients may be intrinsically less susceptible to CA-AKI, making any incremental effect of contrast volume more difficult to detect.

Finally, the low event rate and limited sample size may also have reduced the statistical power to identify a modest effect of contrast volume. Notably, all previous studies of NES have likewise failed to demonstrate a significant association between contrast volume and CA-AKI,^5–13^ except for one study that did not analyze this association.^14^ Therefore, the available evidence suggests that differences in baseline patient risk profiles rather than routine hydration may better explain the absence of a detectable association between contrast volume and CA-AKI in our cohort. Further large-scale studies are therefore needed to clarify this association in NES cohorts.

Among laboratory predictors, lower total protein was independently associated with CA-AKI. Although no prior studies have specifically evaluated total protein, hypoalbuminemia has been linked to CA-AKI in PCI patients, likely reflecting albumin’s roles in maintaining intravascular volume and exerting antioxidant, anti-inflammatory, and toxin-binding effects.^34^ Higher preoperative creatinine also independently predicted CA-AKI, consistent with chronic kidney disease being a risk factor in PCI cohorts^2,3^ and in some NES cohorts.^7,10,13^ Diabetes mellitus was significantly associated with CA-AKI, consistent with findings in PCA cohorts^2,3^ and in some NES cohorts.^7,8,12^

A lack of data from patients undergoing thrombectomy for acute ischemic stroke is one of the major limitations of the present study. However, including these patients would have introduced substantial heterogeneity in data availability, anesthetic management (general vs. local anesthesia), and operator background (neurosurgeons vs. neurologists) in this study’s setting. A meta-analysis of CA-AKI after thrombectomy reported a pooled CA-AKI incidence of 5.0% (95% CI, 2.1%–8.9%), which is substantially higher than that observed in our largest UCA cohort (0.6%). This difference is likely attributable to the older age and higher prevalences of chronic kidney disease, diabetes mellitus, hypertension, and cardiovascular comorbidities, including coronary artery disease and atrial fibrillation, among patients undergoing thrombectomy,^35,36^ compared with those in our UCA cohort.

Limitations of this study include its retrospective, single-center design; the unavailability of postoperative urine output data to improve diagnostic accuracy; unaddressed potential confounders in the analysis, such as perioperative hemodynamic instability and systemic inflammation (especially in CAS and aSAH patients); the small number of CA-AKI events, which limits regression power; and the exclusion of patients undergoing thrombectomy for acute ischemic stroke, the most commonly performed procedure.

## Conclusions

Neuroendovascular procedures for aneurysmal subarachnoid hemorrhage and carotid artery stenosis were associated with an increased risk of contrast-associated acute kidney injury, independent of diabetes mellitus and preoperative total protein and creatinine levels, whereas procedures for other lesions, including unruptured cerebral aneurysm, carried lower risk. To our knowledge, this is the first report demonstrating that risk of contrast-associated kidney injury varies substantially by neuroendovascular procedure type, highlighting the need for tailored preventive strategies.

## Data Availability

The data sets associated with this study are available from the corresponding author upon reasonable request.*
